# Can p63 serve as a biomarker for diagnosing giant cell tumor of bone? A systematic review and meta-analysis

**DOI:** 10.1590/1516-3180.2020.0021.R3.24062020

**Published:** 2020-10-20

**Authors:** Zihao Wan, Chien-Wei Lee, Shuai Yuan, Oscar Kuang-Shen Lee

**Affiliations:** I MD. Doctoral Student, Department of Orthopaedics and Traumatology, The Chinese University of Hong Kong, Hong Kong, China.; II PhD. Research Associate Professor, Institute for Tissue Engineering and Regenerative Medicine, The Chinese University of Hong Kong; and Research Associate Professor, School of Biomedical Science, The Chinese University of Hong Kong, Hong Kong, China.; III MSc. Doctoral Student, Cardiovascular and Nutritional Epidemiology Unit, Institute of Environmental Medicine, Karolinska Institute, Stockholm, Sweden.; IV MD, PhD, Professor, Department of Orthopaedics and Traumatology, The Chinese University of Hong Kong; Professor, Institute for Tissue Engineering and Regenerative Medicine, The Chinese University of Hong Kong; and Professor, Li Ka Shing Institute of Health Sciences, Prince of Wales Hospital, The Chinese University of Hong Kong, Hong Kong, China.

**Keywords:** Biomarkers, Diagnosis, Giant cell tumor of bone, p63, Benign tumor, GCTB

## Abstract

**BACKGROUND:**

Tumor protein p63 (p63) has been reported to be highly expressed in giant cell tumor of bone (GCTB). Whether p63 can be treated as a diagnostic marker for GCTB remains unclear.

**OBJECTIVE:**

We conducted a meta-analysis to evaluate the applicability of p63 in diagnosing GCTB.

**DESIGN AND SETTING:**

Systematic review and meta-analysis carried out in a public hospital, Hong Kong, China.

**METHODS:**

We searched PubMed, EMBASE and the Cochrane Library from inception to April 30, 2019. Literature in English or Chinese about the differential diagnosis of GCTB using p63 were included. ­Animal experiments, reviews, correspondence, case reports, expert opinions and editorials were excluded. Studies were also excluded if they did not provide sufficient information to construct a 2 × 2 contingency table. We calculated individual and pooled sensitivities and specificities. We used I² as an indicator of heterogeneity.

**RESULTS:**

Out of 88 records identified, 8 articles on 788 GCTB patients fulfilled the inclusion criteria and were included in the present analysis. Bivariate analyses yielded a pooled mean sensitivity of 0.87 (95% confidence interval, CI, 0.72-0.95) and specificity of 0.71 (95% CI, 0.56-0.82) for using p63 as a biomarker in diagnosing GCTB. The area under the receiver operating characteristic curve was 0.86 (95% CI, 0.82-0.88).

**CONCLUSION:**

p63 is a helpful indicator in diagnosing GCTB due to its high sensitivity and specificity. ­Nonetheless, the results need to be carefully interpreted based on other diagnostic methods such as imaging.

**SYSTEMATIC REVIEW REGISTRATION:**

164115 (PROSPERO registration number)

## INTRODUCTION

Giant cell tumor of bone (GCTB) is the prototype of giant cell-rich neoplasms of the skeleton, representing 4% to 5% of all primary bone tumors. GCTB mainly occurs in skeletally mature patients, with a peak incidence between ages 20 and 45 years and slight predominance among females.^1^^−^^3^ GCTB commonly arises at the epiphyses of long bones, like the distal femur, proximal tibia, distal radius and proximal humerus.^4^ In addition, it is often found close to joints, and therefore causes movement limitation, joint effusion and synovitis.

At the time of diagnosis, approximately 12% of patients with GCTB present with pathological fractures.^5^^,^^6^ These tumors are locally aggressive with a tendency to recur.^7^^,^^8^ Lung metastases occur infrequently.^9^^,^^10^ The typical appearance of GCTB is best demonstrated on conventional radiographs, which show a lytic lesion that has a well-defined but nonsclerotic margin, is eccentric in location, extends to the subchondral bone and occurs in patients with a closed physis.^11^^−^^13^ The tumor component is heterogenetic. There are mainly three types of cells in the tumor, including osteoclast-like giant cells, macrophage-like cells and stromal cells. Stromal cells are considered to be the neoplastic component of GCTB.^12^^,^^14^^,^^15^

The diagnosis of GCTB is based not only on histology but also on clinical and radiological data.^16^ GCTB is usually a solid mass and brownish in color. Typically, it is characterized by abundant osteoclast-like giant cells surrounded by spindle cells in histological appearance. Usually, a planned biopsy for GCTB is the gold standard for pathological assessment. While the diagnosis is often straightforward, it can be challenging with small core needle biopsies, particularly when dealing with unusual sites or skeletally immature patients.^17^

p63 belongs to the family of transcription factors that also includes p53 and p73.^18^ Giant cells are demarcated through CD63 immunohistochemical staining. This staining basically marks osteoclastic giant cells and macrophages and indicates that these cells originate from the monophagocytic-macrophagocytic system.^18^ It is mostly used as a diagnostic aid in cases of breast, prostate and salivary gland cancer because of its high sensitivity and specificity for mammary and salivary myoepithelial cells and prostatic basal cells.^19^^,^^20^^,^^18^ p63 has also been identified as highly expressed in GCTB, but opinions regarding the usefulness of p63 as a diagnostic marker for the disease have been divergent.^19^^,^^18^

## OBJECTIVE

The objective of this study was to summarizes the current evidence for validation of the diagnostic value of p63 in cases of GCTB.

## METHODS

### Search strategy and selection criteria

We systematically searched PubMed, Embase and the Cochrane Library (from inception to April 30, 2019) for studies assessing the accuracy of p63 as a diagnosis indicator of GCTB. The search strategy is shown in Table 1. We also reviewed the reference lists of each primary study identified and of previous systematic reviews. English and Chinese language restrictions were imposed.

**Table 1:** Search strategy

Database	Search terms	Results
MEDLINE-PubMed (1950-April 30, 2019)	(((((“Giant Cell Tumors”[Mesh]) OR “Giant Cell Tumor of Bone”[Mesh])) AND ((“TP63 protein, human” [Supplementary Concept]) OR P63))) OR (((((giant) AND cell) AND tumor)) AND ((“TP63 protein, human” [Supplementary Concept]) OR P63))	51 studies
EMBASE (1946-April 30, 2019)	giant AND cell AND tumor p63 OR TP63 (1) and (2)	33 studies
Cochrane Library (inception) to April 30, 2019	GIANT and CELL and TUMOR:ti,ab,kw (Word variations have been searched) "p63" or "TP63":ti,ab,kw (Word variations have been searched) (1) and (2)	4 studies

Studies were included if they met following criteria: (1) they assessed the accuracy of p63 for diagnosing GCTB; (2) the gold standard was histological diagnosis; and (3) sufficient information to construct a 2×2 contingency table was provided. Animal experiments, reviews, correspondence, case reports, expert opinions and editorials were excluded.

Data extraction was performed by two reviewers independently. Disagreements were resolved by reaching a consensus or through discussion among the coauthors. The extracted data comprised the general and detailed methodological characteristics, characteristics of the study population, details of the p63 assays and the numbers of true and false positives and negatives.

All studies included in the diagnostic review were assessed for methodological quality using the QUADAS-2 measurement of bias and applicability, by two reviewers, and any disagreements were resolved through reaching a consensus.

### Statistical analysis

We tabulated true positives, false negatives, false positives and true negatives among patients with GCTB, stratified according to study, and calculated the sensitivity and specificity and corresponding confidence interval (CI). To synthetize the data, we used an exact binomial rendition of the bivariate mixed-effects regression model for meta-analyses on treatment trials, with modification for synthesis of diagnostic test data.^21^^−^^24^ This model does not transform pairs of sensitivity and specificity of individual studies into a single indicator of diagnostic accuracy, but it preserves the two-dimensional nature of the data and takes into account any correlation between the two.

We estimated mean logit sensitivity and specificity with their standard error and 95% CIs, the between-study variability in logit sensitivity and specificity, and the covariance. We back-transformed these quantities to the original receiver operating curve scale to obtain summary sensitivity and specificity, and diagnostic odds ratios. We then used the derived logit estimates of sensitivity and specificity, and their respective variances, to construct a hierarchical summary receiver operating curve for p63 with summary operating points for sensitivity and specificity on the curves and a 95% confidence contour ellipsoid (two-dimensional CI).

We calculated I² to assess heterogeneity. If heterogeneity among studies was recorded, the potential source of heterogeneity was investigated through subgroup analysis. To investigate publication bias, we constructed effective sample size funnel plots versus the log diagnostic odds ratio and did a regression test on asymmetry.^25^

The MIDAS module 22 was used in the bivariate summary receiver operating curve analysis. We used the MIDAS module and the Quality Assessment of Diagnostic Accuracy Studies module to evaluate the quality of the studies included. All analyses were performed in the STATA software (version 15.1, StataCorp, Texas, United States).

## RESULTS

**Figure 1:**
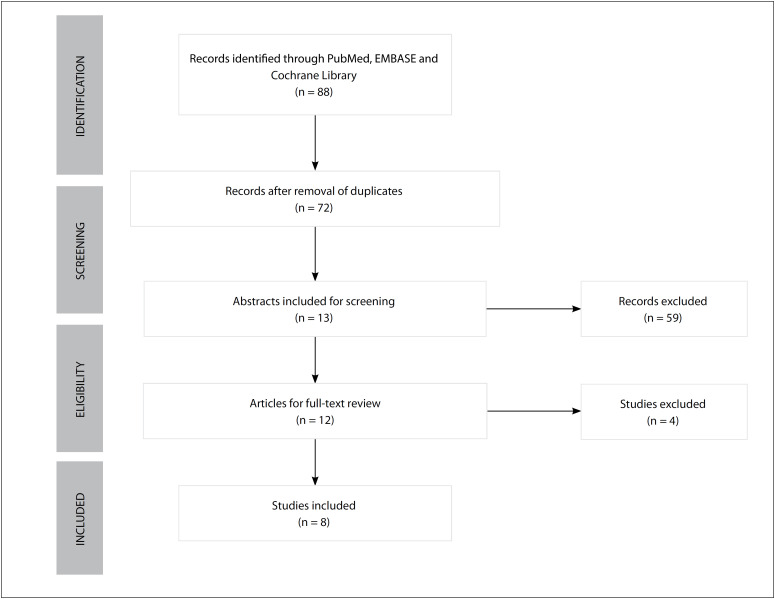
Study selection process.

**Figure 2:**
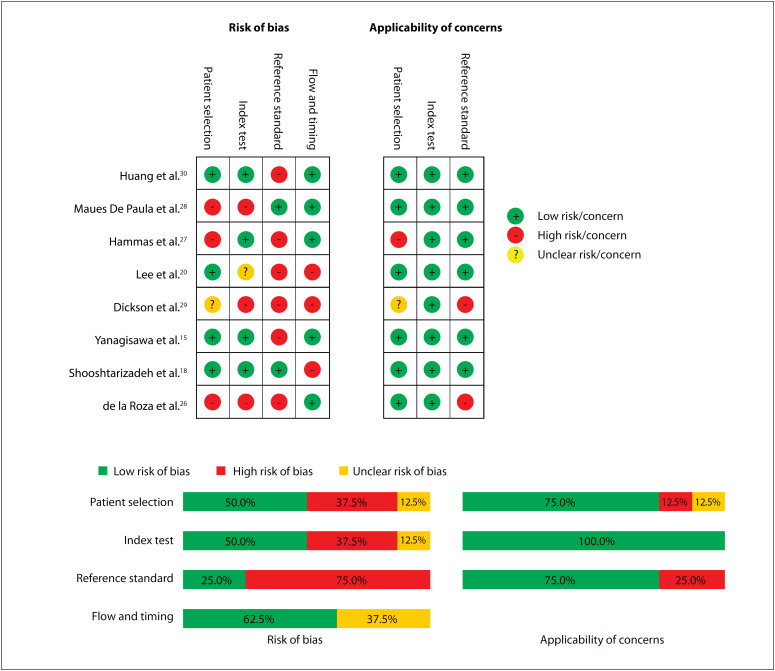
Quality assessment.

Out of the 88 articles retrieved, 76 papers were excluded after duplicates, titles and abstracts had been assessed. We further excluded four papers after full-text reviewing, thus leaving eight studies in the present analysis (Figure 1). The result from the quality assessment is shown in Figure 2.^15^^,^^18^^,^^20^^,^^26^^−^^30^

Table 2 shows the characteristics of the eight studies included. In total, 788 critically ill patients were included in the analysis, of whom 335 (42.5%) suffered from GCTB. The prevalence of GCTB among the studies ranged from 6.6% to 86.8% (mean of 42.5%).^15^^,^^18^^,^^20^^,^^26^^−^^30^

**Table 2:** Diagnostic accuracy results

Authors	Year	n	TP	FN	Sensitivity (95% CI)	FP	TN	Specificity (95% CI)
Huang et al.^30^	2014	136	99	19	0.84 (0.76-0.90)	3	15	0.83 (0.59-0.96)
Maues De Paula et al.^28^	2014	272	98	21	0.82 (0.74-0.89)	72	81	0.53 (0.45-0.61)
Hammas et al.^27^	2012	48	5	0	1.00 (0.48-1.00)	20	23	0.53 (0.38-0.69)
Lee et al.^20^	2008	91	5	1	0.83 (0.36-1.00)	13	72	0.85 (0.75-0.92)
Dickson et al.^29^	2008	46	17	0	1.00 (0.80-1.00)	5	24	0.83 (0.64-0.94)
Yanagisawa et al.^15^	2013	36	6	10	0.38 (0.15-0.65)	2	18	0.90 (0.68-0.99)
Shooshtarizadeh et al.^18^	2016	100	30	1	0.97 (0.84-1.00)	24	45	0.65 (0.53-0.76)
de la Roza^26^	2011	59	20	3	0.87 (0.66-0.97)	22	14	0.39 (0.23-0.57)
**Total**		**788**	**280**	**55**		**161**	**292**	

TP = true positive; FN = false negative; FP = false positive; TN = true negative; CI = confidence interval.

**Figure 3:**
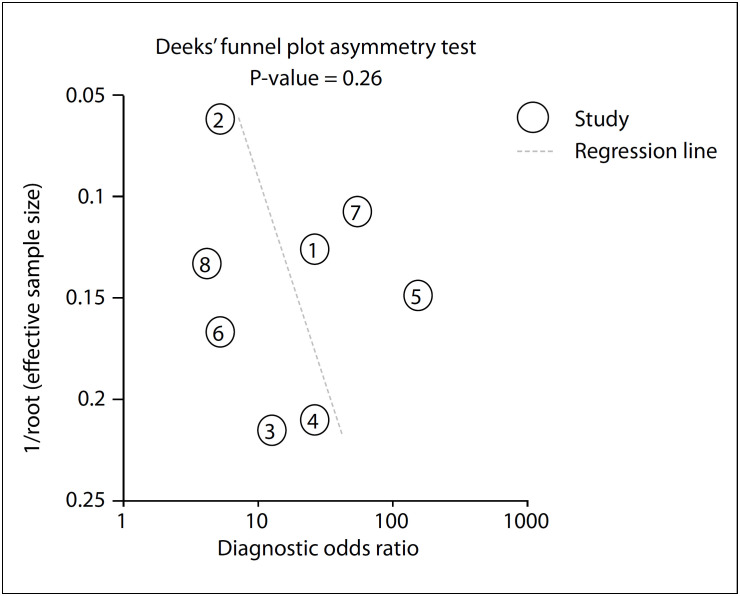
Deeks’ funnel plot asymmetry test for publication bias.

**Figure 4:**
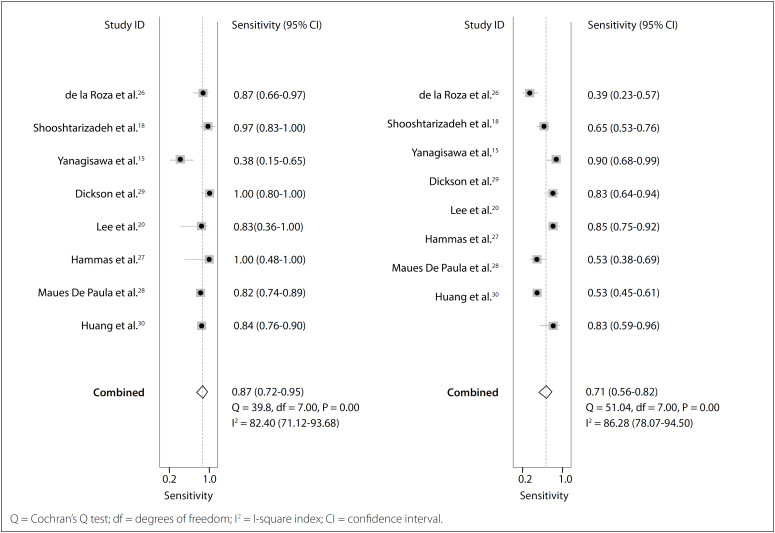
Sensitivity and specificity of p63 test for diagnosis of giant cell tumor of bone (GCTB).

**Figure 5:**
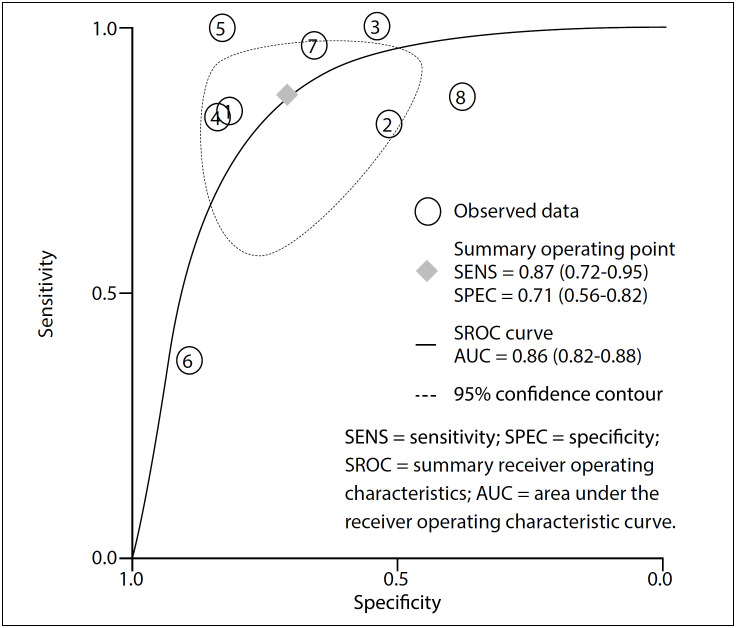
Summary receiver operating characteristic curve.

No publication bias was identified through Deeks’ regression test of asymmetry (t = 1.24; P = 0.26; Figure 3).^15^^,^^18^^,^^20^^,^^26^^−^^30^ The pooled sensitivity of p63 was 0.87 (95% CI, 0.72-0.95) and the specificity was 0.71 (95% CI, 0.56-0.82), as an indicator in making the diagnosis of GCTB (Figure 4). The area under the receiver operating characteristic curve was 0.86 (95% CI, 0.82-0.88) (Figure 5). We detected substantial significant heterogeneity among the studies included (overall I², 90%; 95% CI, 80-100). The samples included were stratified according to gender, age range, complications and lesion sites, if information relating to these factors was available. However, no subgroup analysis could explain the significant heterogeneity.

**Figure 6:**
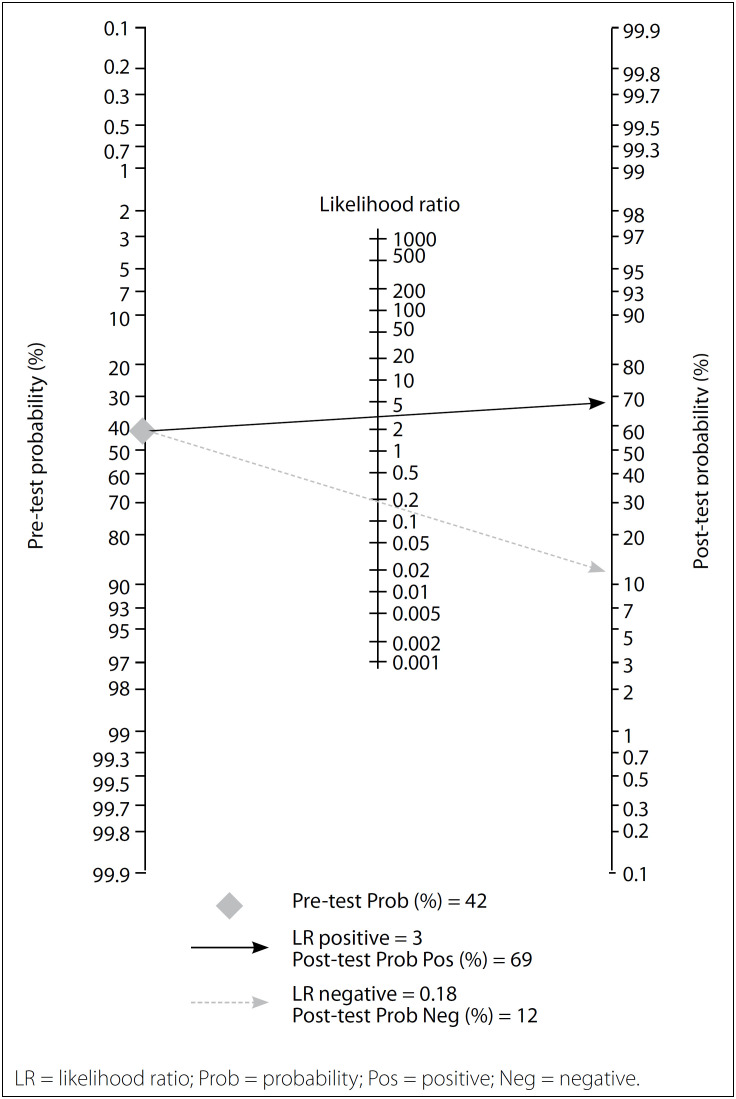
Fagan nomogram of the p63 test for diagnosis of giant cell tumor of bone (GCTB).

In our study, both the likelihood ratio and the post-test probability were moderate (Figure 6).

Given a pretest probability of 42%, the post-test probability for a positive test result is 69%. Likewise, a negative likelihood ratio of 0.18 reduces the post-test probability to 12% for a negative test result.

## DISCUSSION

There are multiple giant-cell-rich bone tumors that can express p63, although the expression level varies. However, there is no consensus regarding the p63 expression level of GCTB clinically.^1^^,^^18^^,^^20^ Researchers or clinicians have proposed that a certain percentage of p63 expression in giant cells can be used as a cutoff value in making the diagnosis of GCTB.^28^ Maues De Paula et al.^28^ declared that a finding of more than 50% of the cells positive for p63 was highly related to a diagnosis of GCTB while percentages lower than 50% appeared to be nonspecific. Nevertheless, we are unable to define a cutoff value for p63 expression levels because of discrepancies in the standards used for evaluating p63 expression between the different studies.

Likelihood ratios and post-test probabilities are also relevant for clinicians. They provide information about the likelihood that a patient with a positive or negative test actually has GCTB or not. A certain positive likelihood ratio indicates that a person with disease is a certain number of times more likely to have a positive test result than is a healthy person. However, these likelihood ratios are calculated from dichotomized data. The result from the p63 test is either positive or negative. The disadvantage of making data dichotomous is that useful information is lost.^31^ Because p63 expression levels rise as disease severity advances, patients with a high p63 expression level are more likely to be diagnosed with GCTB than are patients with a low p63 expression level.^15^ To provide more precise information about the reliability of the test, we suggest that likelihood ratios should be calculated based on multiple cutoffs.

As our results show, p63 is not a single definitive diagnostic marker for diagnosing GCTB. GCTB is a pathophysiological process rather than a specific syndrome and is too complex to be described through a single measurement. Nevertheless, p63 is one of the most promising parameters.

There are several limitations to the present meta-analysis. First, we detected substantial heterogeneity between studies. However, subgroup analysis did not find any source of heterogeneity. The unrecorded differences between the studies probably contributed to the heterogeneity. Second, a reliable test for infection is still under investigation, so observational studies are biased through the choice of gold standard. Third, most of the studies included did not provide detailed information about the treatments received, disease stages and recurrence situation. Absence of detailed patient histories could cause interobserver variability, which could lead to false-negative or false-positive judgments about the patient's medical condition. Lastly, we only included studies published in English, which also may potentially have caused bias through the language restriction in this specific systematic review.

## CONCLUSION

p63 is a helpful marker for diagnosing GCTB in critically ill patients. However, it cannot be recommended as the single definitive test for making this diagnosis. The results need to be carefully interpreted in conjunction with other diagnostic methods such as imaging studies. Moreover, continuing re-evaluation of p63 during the course of the disease is warranted.
